# Effects of high night temperature on soybean yield and compositions

**DOI:** 10.3389/fpls.2023.1065604

**Published:** 2023-02-17

**Authors:** Lin Yang, Wenwen Song, Cailong Xu, Enoch Sapey, Dong Jiang, Cunxiang Wu

**Affiliations:** ^1^ Key Laboratory of Crop Physiology and Ecology in Southern China, Ministry of Agriculture, Nanjing Agricultural University, Nanjing, China; ^2^ Ministry of Agriculture and Rural Affairs Key Laboratory of Soybean Biology (Beijing), Institute of Crop Sciences, Chinese Academy of Agricultural Sciences, Beijing, China; ^3^ Council for Scientific and Industrial Research (CSIR), Oil Palm Research Institute, Kade, Ghana

**Keywords:** high night temperature, soybean, yield, carbohydrates, transcriptome

## Abstract

**Introduction:**

Soybean is sensitive to light and temperature. Under the background of global asymmetric climate warming.

**Methods:**

The increase of night temperature may have an important impact on soybean yield. In this study, three varieties with different level of protein were planted under 18°C and 28°C night temperatures for investigating the effects of high night temperatures on soybean yield formation and the dynamic changes of non-structural carbohydrates (NSC) during the seed filling period (R5-R7).

**Results and discussion:**

The results indicated that high night temperatures resulted in smaller seed size, lower seed weight, and a reduced number of effective pods and seeds per plant, and thus, a significant reduction in yield per plant. Analysis of the seed composition variations showed carbohydrates were more substantially affected by high night temperature than protein and oil. We observed “carbon hunger” caused by high night temperature increased photosynthesis and sucrose accumulation in the leaves during the early stage of high night temperature treatment. With elongated treated time, the excessive carbon consumption led to the decrease of sucrose accumulation in soybean seeds. Transcriptome analysis of leaves after 7 days of treatment showed that the expression of most sucrose synthase and sucrose phosphatase genes decreased significantly under the high night temperature. Which could be another important reason for the decrease of sucrose. These findings provided a theoretical basis for enhancing the tolerance of soybean to high night temperature.

## 1 Introduction

Soybean (*Glycine max*) is one of the most important food crops. One of the major compositions in soybean seeds is protein, which is necessary for human nutrition; soy is also a valuable source of edible oil (Medic et al., 2014). During the developmental stage, soybeans are often at risk of high temperature stress. The Intergovernmental Panel on Climate Change (IPCC) reports that future climate change will expose crops to heightened average temperatures and increase the frequency of short-term high temperatures (Solomon, 2008). High temperature causes significant agricultural losses, affects nutrient cycling in many crops, and leads to substantial yield decline (Asseng et al., 2014, Schauberger et al., 2017; Zhao et al., 2017). When soybeans suffer high temperatures during the seed filling stage, their yields are reduced and their seed compositions changed (Medic et al., 2014; Song et al., 2016; Nakagawa et al., 2020). For legumes such as faba beans, various genetic approaches have been used to breed for heat stress tolerance varieties (Lavania et al., 2015; Lavania et al., 2016). Global climate model projections indicate that nighttime warming rises more rapidly than daytime warming and is the primary driver of global warming (Davy et al., 2016), reducing diurnal temperature differences (Vose et al., 2005). A study by our research group found that the diurnal temperature difference had a greater effect on soybean protein and oil contents relative to the average daily temperature, suggesting that night temperature plays an important role in soybean protein and oil accumulation (Song et al., 2016). Therefore, studying the effects of nighttime warming on soybean growth and yield will provide a theoretical basis for developing future food security strategies in China.

Several studies have confirmed the effects of temperature on soybean seed growth in different developmental stages, duration of high temperature treatments, and intensities of temperatures (Gibson, 1992; Tacarindua et al., 2013; Nakagawa et al., 2020). The impact of high temperatures on soybean yield and quality has been studied using open-enclosure facilities, controlled artificial climate chambers, or crop models based on historical data (Gibson, 1992; Tacarindua et al., 2013; Nakagawa et al., 2020). A negative correlation between soybean yield and seasonal warming has been discovered (Kucharik and Serbin, 2008). For every 1°C increase, soybean yield decreases by an average of 17% (Lobell and Asner, 2003). The high temperature associated with damage to soybean growth and development took place in controlled chambers and greenhouses (Gibson, 1992; Tacarindua et al., 2013; Nakagawa et al., 2020). Some studies reported a decrease in yield at high temperatures in the soybean seed filling stage (Gibson and Mullen, 1996; Siebers et al., 2015). It was observed that high temperature stress experienced during mid-reproductive growth is more detrimental to seed yield and seed size than that experienced during early or late reproductive development stage (Puteh et al., 2013). Extreme heat or heat stress can cause irreversible damage to soybean growth and development, and can even cause death (Feng et al., 2020). The moderately high temperatures are more likely to lead to morphological, structural, physiological, and biochemical changes in the thermal adaptation to warming conditions (Puteh et al., 2013). It was found that moderate high night temperature of 29°C reduced photosynthetic rates and pollen germination, resulting in decreased pod numbers and seed weight (Djanaguiraman et al., 2013). However, the effect of high night temperature, seed’s C and N accumulation, and corresponding underlying mechanisms in soybeans remains unclear.

It has been suggested that high night temperature stimulate carbohydrate usage (Loka and Oosterhuis, 2010). The yield decline is mainly due to a reduction in the biomass allocated to the coffers and a decrease in the content of dry matter and its synthetics. Losses caused by high night temperature are usually accompanied by a decline in biomass accumulation (Zhang et al., 2013; Jing et al., 2016). Daytime is a crucial period for photosynthesis in soybeans; at night, the plants turn into mere consumers of the bioproducts of photosynthesis, which accumulate during the day (Xu et al., 2021). Many studies have confirmed that respiration rates of many crops such as rice (Alvarado-Sanabria et al., 2017), wheat and barley (García et al., 2015), maize (Kucharik and Serbin, 2008), and cotton (Loka and Oosterhuis, 2010) increase at high night temperature. The same result was also reported for soybeans (Bunce, 2005; Djanaguiraman et al., 2013; Lin et al., 2021). As plants enhance nocturnal respiration (Rn), more photosynthetic bioproducts are consumed, thereby reducing the sugar reserves available for seed filling (Xu et al., 2021), night temperatures of 10°C above ambient lead to a 2.6-fold enhancement in rapid nocturnal respiration. However, the response diminishes after thermal acclimation and causes yield loss (Peraudeau et al., 2015). It was reported that the high night temperature resulted in the higher Rn and decline of the yield in soybean by 4.6% per °C (Lin et al., 2021). Another study found that various soybean varieties showed different level of tolerance to high temperature, only a few varieties showed decrease yield under high night temperature (Shu, 2021). One study has also observed that high night temperature causes many environmental variables and that respiration has no effect on yield or biomass (Frantz et al., 2004).

Non-structural carbohydrates in leaves, such as sucrose and starch, represent the state of photosynthesis in the leaf, provide whole-plant energy, and contribute to the accumulation of structural carbohydrates (Hussain et al., 2019; Stitt et al., 1987). Leaf senescence may affect crop yield in two different ways. On one hand, delayed leaf senescence can increase yield by prolonging photosynthesis and extending seed filling period; On the other hand, delayed leaf senescence may affect nutrient reuse to the sink organ, which may harm yield (Guo et al., 2021). Sucrose is the main source of carbon energy for developing seeds and sucrose transport is the central system of carbon resource allocation for the whole plant (Ruan, 2014; Petridis et al., 2020; Zhang et al., 2016). It has been reported that sucrose content decreases with the increase in temperature (Loka and Oosterhuis, 2010). Studies have shown proteomic changes in different tissues like leaves and anthers of plants at high temperatures, as well as differential expression of proteins related to various physiological processes, such as carbohydrate metabolism (Das et al., 2015; Kumar et al., 2022; Zhou et al., 2017). The balance between sucrose synthesis and catabolism in leaves is mediated by influencing the activity of sucrose synthase (SS) and sucrose phosphate synthase (SPS), two key enzymes that also control the biosynthesis of carbon into starch (Stitt and Zeeman, 2012) However, few researchers have studied the effect of night temperature on the biosynthesis of leaf sucrose.

Currently, RNA-seq technology is widely used in temperature regulation studies in plants (Gillman et al., 2019; Shu et al., 2020). RNA-Seq analysis of whole wheat kernels showed that high temperature induced down-regulation of genes involved in regulating pericarp cell wall expansion (Kino et al., 2020). This down-regulation coincided with a reduction in total grain water content and weight after the same treatment period, supporting the theory that high temperature may lead to a reduction in mature grain weight (Kino et al., 2020). Proteins like heat shock proteins also known to play a key role in leguminous plants like *Vicia faba* and other plants under heat stress (Kumar et al., 2015; Kumar et al., 2016; Kumar et al., 2020; Tiwari et al., 2021).

The aims of the study were (i) to evaluate the effect of high night temperature during the seed filling stage on the yield and seed compositions, (ii) to discover the dynamic changes of photosynthetic physiological characteristics, dry matter accumulation and NSC linked to stress tolerance triggered by high night temperature, and (iii) to analyze the molecular basis of changes in the leaves, specifically sugar metabolism.

## 2 Materials and methods

### 2.1 Experimental site and materials

The experiment was conducted for two years (2020-2021) at the Photothermal Control Complex at Changping Experimental Station (Beijing, 40°18′ N,116°25′ E), Institute of Crop Sciences, Chinese Academy of Agricultural Sciences.

The high-protein variety Zhonghuang 39 (ZH39), medium-protein variety Zheng 1307 (Z1307), and low-protein variety Zhonghuang 76 (ZH76), which have the same growth period traits (belonging to MG III, with similar flowering time and growth period), were selected as experimental materials. All the materials were planted in pots with dimensions of 18.5 cm in height and 18 cm in diameter and filled with 10 kg of soil. Ten seeds were sown in each pot on June 24th, and the plants were thinned to five healthy and uniform plants in each pot after emergence. All the plants were grown to the beginning of seed stage (R5) under natural light and temperature conditions, and good agronomic practices were carried out. From the stage of R5 to R7 (physiological maturity), the materials were treated with different night temperature.

### 2.2 Experimental design and management

Four temperature-controlled chambers (6m×3m×2.5m) were used to study the impact of high night temperature. According to the average daily and nightly temperatures (28°C and 18°C) and the sunlight duration (12.5 h) during seed filling stage (September) in Beijing, all the plants were exposed to 18°C or 28°C night temperature with a natural day temperature and 12-h light (7 pm - 7 am) treatment. Each night temperature treatment corresponded to a chamber installed with two platform trailers, and the pots were placed on the trailers during the experimental period. During the day time, the trailers were pushed outside the chambers, and all the plants grew under the natural light and day temperature, and at night they were pushed into the chambers with different temperature treatment.

Temperature and humidity data were recorded every 30 min from the seed filling stage to the maturity stage using a temperature and humidity recorder (OM-EL-WIFI-TH, OMEGA Engineering, USA). The temperature changes during the treatment period (R5-R7) are shown in **Figure S1**. The average night temperature of 18 °C treatment during the period in 2020 was 18.06 °C, with a minimum of 17.36 °C and a maximum of 21.52 °C. The average temperature of the 28 °C treatment was 27.16 °C, with a minimum of 25.21 °C and a maximum of 28.79 °C. During the treatment period in 2021, the average temperature of the 18 °C treatment temperature was 18.29°C, with a minimum of 17.36°C and a maximum of 21.52°C. The average temperature of 28°C treatment was 27.53°C, with a minimum of 25.21°C and a maximum of 28.79°C. Any observed differences were acceptable for the single-night temperature control, and the differences between temperature treatments remained constant, making the two years of experimental condition control reliable.

### 2.3 Seed size

We randomly selected pods grown in similar positions for sampling and analysis. We peeled and photographed the seeds after they reached maturity. Vernier calipers were used as a scale, and LED light was used to remove the soybean seeds’ shadows, in order to get as clear an image as possible. Each seed’s basic dimensions and thickness were measured using a standard vernier caliper (Baek et al., 2020). Each measurement was repeated five times.

### 2.4 Dry matter, sucrose and starch content

Three plants were cut at the cotyledon scars so that aboveground parts could be extracted every 7 days at 9 am - 11 am. Different organs of the plants were separated and placed in an oven at 105°C for 30 minutes for dehydration, then dried at 70°C to constant mass, and weighed. Keming Sucrose Content Test Kit (ZHT-1-Y, Keming, China) and Keming Starch Content Test Kit (DF-1-Y, Keming, China) were used for sucrose and starch content determinations. Each sample was analyzed five times.

### 2.5 Net photosynthesis and SPAD

The net photosynthetic rate of leaves was measured at the same time using a Li-6400 portable photosynthesizer (LI-COR 6400, Lincoln, USA) with the following parameters set: light quantum flux at 1200 umol/(m^2^·s), CO_2_ concentration at 450 umol/m^2^, flow rate at 500 umol/s, and temperature at 25°C. Ten plants were measured during each treatment. The leaf chlorophyll content, denoted by the SPAD value, was measured on a SPAD meter (Chlorophyll Meter Model SPAD-502, Konica Minolta Inc, Japan) every 7 days at 9-11 am after different night temperature treatments (Ergo et al., 2018).

### 2.6 Agronomic traits

Ten mature plants were taken to investigate yield-related agronomic traits (plant height, number of nodes, number of pods per plant, number of seeds per plant, and 100-seed weight) for each treatment. Protein and oil contents of seeds were determined using near-infrared spectroscopy NIR (MPA, Bruker Optics, Germany), and the sum of protein and oil content were calculated. Any remaining fractions (primarily carbohydrates) were labeled ‘residual’ (Tamagno et al., 2022).

### 2.7 RNA-seq (transcriptome) and data analysis

The middle leaves of the third ternately compound leaves from the top of ZH39 at 7 DAT (about 30 days after flowering and 77 days after planting) were collected and flash-frozen with liquid nitrogen, and stored at -80°C freezer for transcriptome analysis. For each treatment, three leaves were sampled and pooled as one biological replicate, and three biological replicates were used. According to the method described by Metware Biotechnology Co. Ltd. (Wuhan, China), total RNA was extracted using RNAprep Pure Plant Plus Kit (DP441, Tiangen, China) according to its protocol. Before library construction, the RNA quality was assessed using the NanoPhotometer spectrophotometer (IMPLEN, CA, USA), Qubit 2.0 Fluorometer (Life Technologies, CA, USA), and Agilent Bioanalyzer 2100 system (Agilent Technologies, CA, USA). Then cluster generation, the library preparations were sent to the llumina Novaseq6000 system for sequencing. Clean data (clean reads) were obtained by removing reads with adapter reads containing ploy-N and low quality reads from raw data. Gene expression levels were determined using the fragments per kilobase of transcript per million reads (FPKM) to compare among different samples. The thresholds for differentially expressed genes (DEGs) were defined by *p* < 0.05 and log_2_ fold ≥ 1 or ≤ -1.

The RNA-seq reads used for this study were deposited at the National Center for Biotechnology Information under project SUB12180172, and the accession number was SAMN31361599-31361604.

### 2.8 Data analysis

Data were entered using Excel 2019 software (Microsoft Corporation, USA) and plotted using Graphpad 8.0.2 (San Diego, USA) and Rstudio 4.20 (R team 2021, USA). Analysis of variance (ANOVA) was done using DPS 7.05 statistical software (Data Processing System, China). Duncan’s multiple comparisons tested the significant differences between means (*P* < 0.05). Other statistical analyses were performed using SPSS 22.0 software (IBM, USA).

## 3 Results and analysis

### 3.1 Seed yield and yield components

High night temperature induced significantly (*P* < 0.01) lower yield in all the three varieties compared (**Table 1**) in 2020 and 2021. In 2020, the yield of ZH39 decreased the most by 33.69%, while the yield of ZH76 dropped the most by 45.45% in 2021. It was shown that the yield components of number of pods per plant, seed number per plant, 100-seed weight, and seed yield per plant completely dropped in various degree under high night temperature condition. The interaction between year and variety effected the 100-seed weight and yield significantly. The interaction between year and treatment showed significant differences in the number of seeds per plant. The interaction of variety and treatment showed significant differences in effective pod number per plant and highly significant differences in seed number per plant. The year, variety, and treatment interaction showed significant differences in effective pods per plant, 100-seed weight, and yield.

**Table 1:** Effects of different high night temperature on yield and components of soybean.

Year	Cultivars	Treatment (°C)	Effective pods number per plant	Seed number per plant	100-seed weight (g)	Yield(g/plant)
2020	Z1307	18	26.67±0.33aA	62.00±1.73aA	16.31±0.06aA	8.41±0.21aA
		28	21.00±1.73bA	28.33±1.45bB	15.40±0.17bB	7.32±0.29bA
	ZH39	18	16.00±0.58aA	41.67±2.33aA	21.98±0.10aA	8.40±0.57aA
		28	10.33±1.86bA	32.67±0.88bA	19.04±0.13bB	5.57±0.35bA
	ZH76	18	22.00±1.53aA	47.33±5.24aA	18.86±0.16aA	7.97±0.35aA
		28	15.33±0.67bA	25.00±1.00bA	17.79±0.07bB	5.96±0.07bB
2021	Z1307	18	34.00±1.00aA	65.67±2.73aA	15.92±0.09aA	9.28±0.29aA
		28	21.33±0.33bB	31.33±4.81bB	13.45±0.07bB	6.12±0.28bB
	ZH39	18	20.33±0.88aA	42.67±2.19aA	22.24±0.11aA	8.09±0.03aA
		28	17.00±0.58bA	22.67±1.20bB	19.69±0.76bA	6.61±0.21bB
	ZH76	18	26.67±1.86aA	55.33±5.67aA	18.40±0.42aA	6.93±0.19aA
		28	20.67±0.33bA	18.00±0.58bB	15.14±0.53bB	3.78±0.28bB
F-value						
Year(Y)			53.21**	NS	18.02**	7.63**
Cultivar(C)			75.24**	18.55**	316.34**	31.38**
Treatment(T)			103.60**	225.18**	152.11**	183.77**
Y*C			NS	NS	11.95**	12.24**
Y*T			NS	6.52*	9.97**	NS
C*T			4.29*	11.61**	NS	NS
Y*C*T			4.81*	NS	4.78*	9.10**

*, ** represent the test significant at the 5% level (P < 0.05) and 1% level (P < 0.01), respectively; NS, test non-significant at the 5% level.

### 3.2 Seed compositions

**Figure 1:**
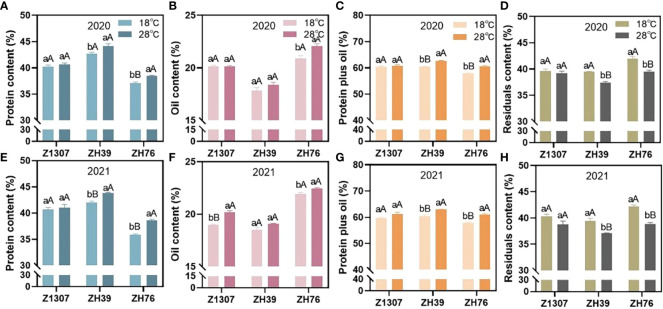
Effect of high night temperature on protein, oil, protein plus oil and residual contents in mature soybean seeds. The soybean seed protein (A-2020, E-2021), oil (B-2020, F-2021), protein plus oil content (C-2020, G-2021) and residual (D-2020, H-2021) contents of the three varieties Z1307, ZH39 and ZH76 under 18°C and 28°C night temperature during seed-filling stage were showed in the figure. The different letters indicate significant difference at 0.05 level (small letter) and 0.01 level (big letter).

The protein, oil, and residue contents in soybean seeds were affected in various degree by the high night temperature in three varieties (**Figure 1**). Under high night temperature, the protein content of ZH76 significantly increased in 2020 (3.69%) and 2021 (7.87%), as well as ZH39 (3.95%) in 2021. However, the protein content of Z1307 showed insignificant change under high night temperature condition in both growing seasons. The oil content of ZH76 significantly increased in 2020 (5.59%) and 2021 (2.31%), as well as Z1307 (6.21%) in 2021 under high night temperature. While, the oil content of ZH39 showed insignificant change in both two years. For the total protein plus oil, ZH76 and ZH39 showed a significant increase average by 5.07% and 3.72% in 2020 and 2021 under the high night temperature, while non-significant difference was found in Z1307 between the treatments. The residual content in ZH39 and ZH76 significant decreased by 5.69% and 6.97% on average under high night temperature condition, respectively. However, the residual content of Z1307 showed non-significant change under high night temperature in both two years.

### 3.3 Dry matter accumulation

**Figure 2:**
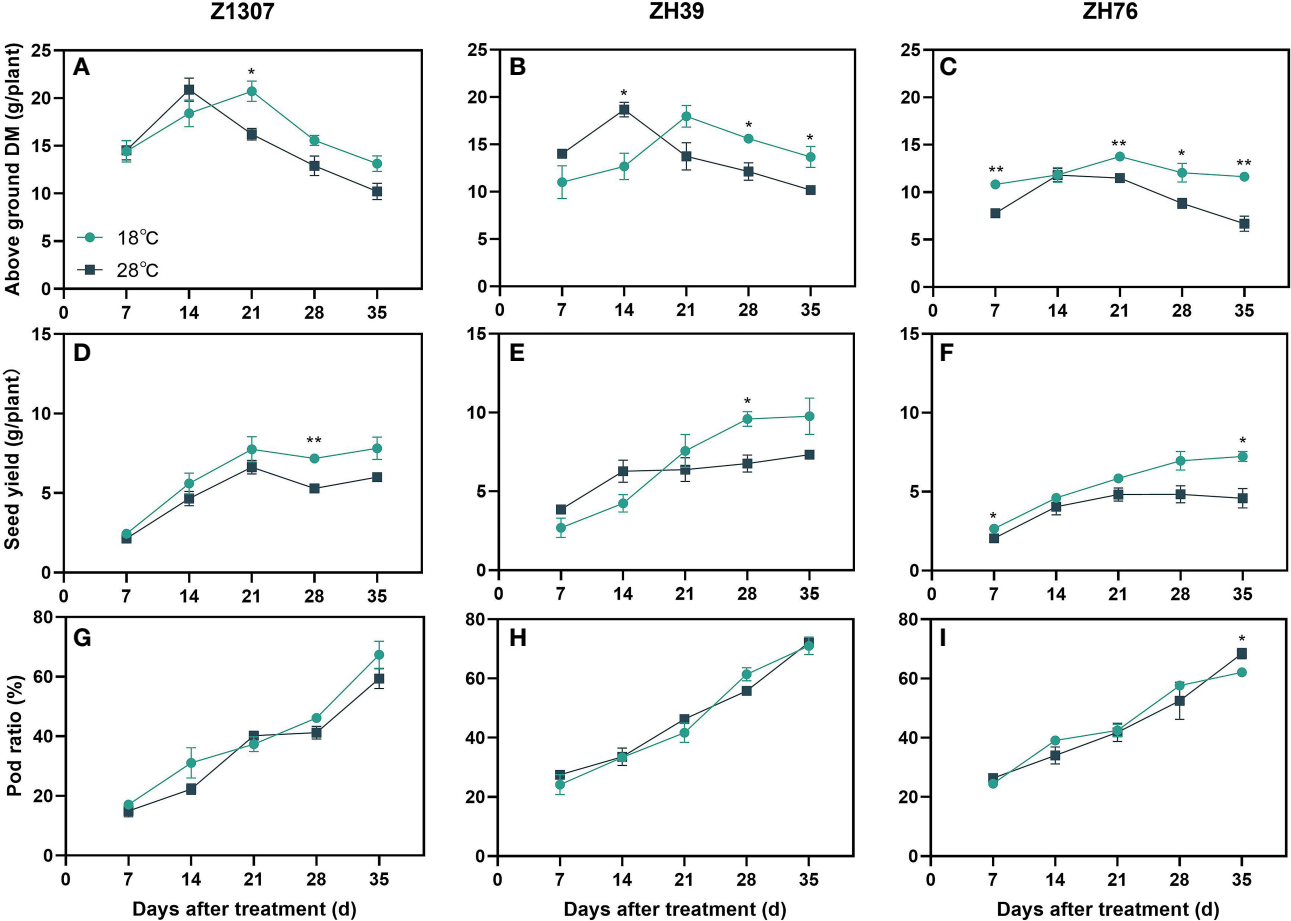
Effects of high night temperature on total dry matter(DM), seed yield and pod ratio. The light blue /dark blue line indicates the 18 ℃/28 ℃ treatment. DM means dry matter. The broken lines respect the dynamic changes of soybean above ground DM **(A-C)**, seed yield **(D-F)** and pod ratio **(G-I)** with the days of different high night temperature treatments for the three varieties of Z1307, ZH39 and ZH76. Values shown are means ± SE from three biological replicates. (*,*P* < 0.05; **, *P* < 0.01).

The aboveground dry matter accumulation of the three varieties showed the same trend of increasing during the 14 DAT and then decreased (**Figures 2A-C**). The peak of aboveground dry matter accumulation appeared at 21 DAT for the 18°C night temperature treatment and 14 DAT for the 28°C night temperature (**Figures 2D-F**). The seed yield per plant of ZH39 was higher under high night temperature before 21 DAT at 28°C night temperature treatment, and it was lower after 21 DAT. For Z1307 and ZH76, the seed yield per plant was consistently lower at 28°C night temperature comparing with 18°C night temperature. No significant differences in the pod ratio were found among the treatments in the three varieties (**Figures 2G-I**).

### 3.4 Seed size

**Figure 3:**
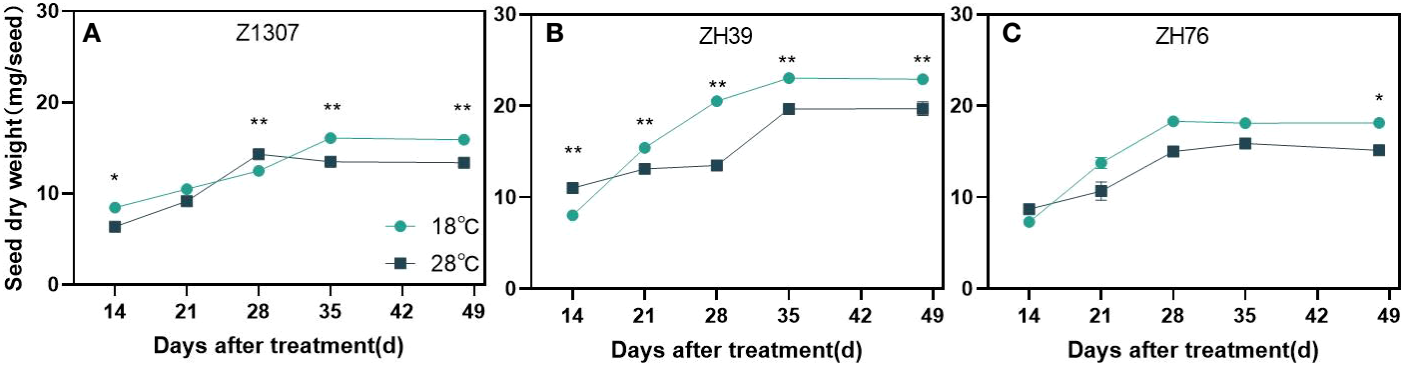
Dynamic changes of soybean seed weight under different night temperatures. The dark blue/black blue line indicates the 18 ℃/28 ℃ treatment. The broken lines respect the dynamic changes of soybean seed weight of the variety Z1307 **(A)**, ZH39 **(B)** and ZH76 **(C)** under different night temperatures. Values shown are means ± SE from three biological replicates. (*,*P* < 0.05;**, *P* < 0.01).

High night temperature had a detrimental effect on seed size in the three varieties (**Figure 3** and **Figure S2**). Generally, the length, width and diameter of seeds showed a significant decrease under high night temperature in the two years (**Table 2**). Among the traits, the length of the seed was mostly affected. The length of Z1307 was reduced by 12.8% and 9.4% in 2020 and 2021, and it was reduced by 11.3% and 16.1% in ZH76. The seed of ZH39 also showed a slight decrease in the length. The seed-length-to-width ratio did not change significantly between the two treatments.

**Table 2:** Effects of different night temperature on seed size of soybean.

Year	Cultivars	Treatment(°C)	Seed length (mm)	Seed width (mm)	Seed diameter (mm)	Ratio of seed length to width
2020	Z1307	18	7.57±0.07aA	6.23±0.03aA	6.73±0.07aA	1.21±0.02aA
		28	6.60±0.00bB	5.63±0.07bB	6.07±0.03bB	1.17±0.01aA
	ZH39	18	7.83±0.03aA	6.50±0.06aA	6.93±0.18aA	1.21±0.01aA
		28	7.33±0.09bB	6.27±0.07aA	6.53±0.03aA	1.17±0.02aA
	ZH76	18	7.70±0.06aA	5.97±0.12aA	6.67±0.03aA	1.29±0.02aA
		28	6.83±0.07bB	5.58±0.02bA	6.37±0.09bA	1.22±0.01aA
2021	Z1307	18	7.23±0.13aA	6.40±0.09aA	6.73±0.02aA	1.13±0.03bA
		28	6.55±0.12bA	5.10±0.06bB	5.71±0.22bA	1.28±0.03aA
	ZH39	18	7.96±0.05aA	6.51±0.15aA	7.01±0.10aA	1.22±0.02aA
		28	7.57±0.07bB	5.95±0.08bA	6.70±0.02bA	1.27±0.02aA
	ZH76	18	8.27±0.10aA	5.56±0.08aA	6.89±0.06aA	1.49±0.04aA
		28	6.95±0.17bB	4.96±0.22aA	6.05±0.15bB	1.41±0.06aA
F-value						
Year(Y)			4.48*	23.29**	NS	26.17**
Cultivar(C)			60.77**	61.33**	21.78**	33.15**
Treatment(T)			229.04**	109.99**	94.67**	NS
Y*C			9.46**	3.87*	NS	9.52**
Y*T			NS	12.49**	4.82*	6.80*
C*T			13.02**	8.62**	5.46*	5.04*
Y*C*T			4.56*	NS	NS	NS

*, ** represent the test significant at the 5% level (*P* < 0.05) and 1% level (*P* < 0.01), respectively; NS, non-significant at the 5% level.

### 3.5 Photosynthetic characteristics

**Figure 4:**
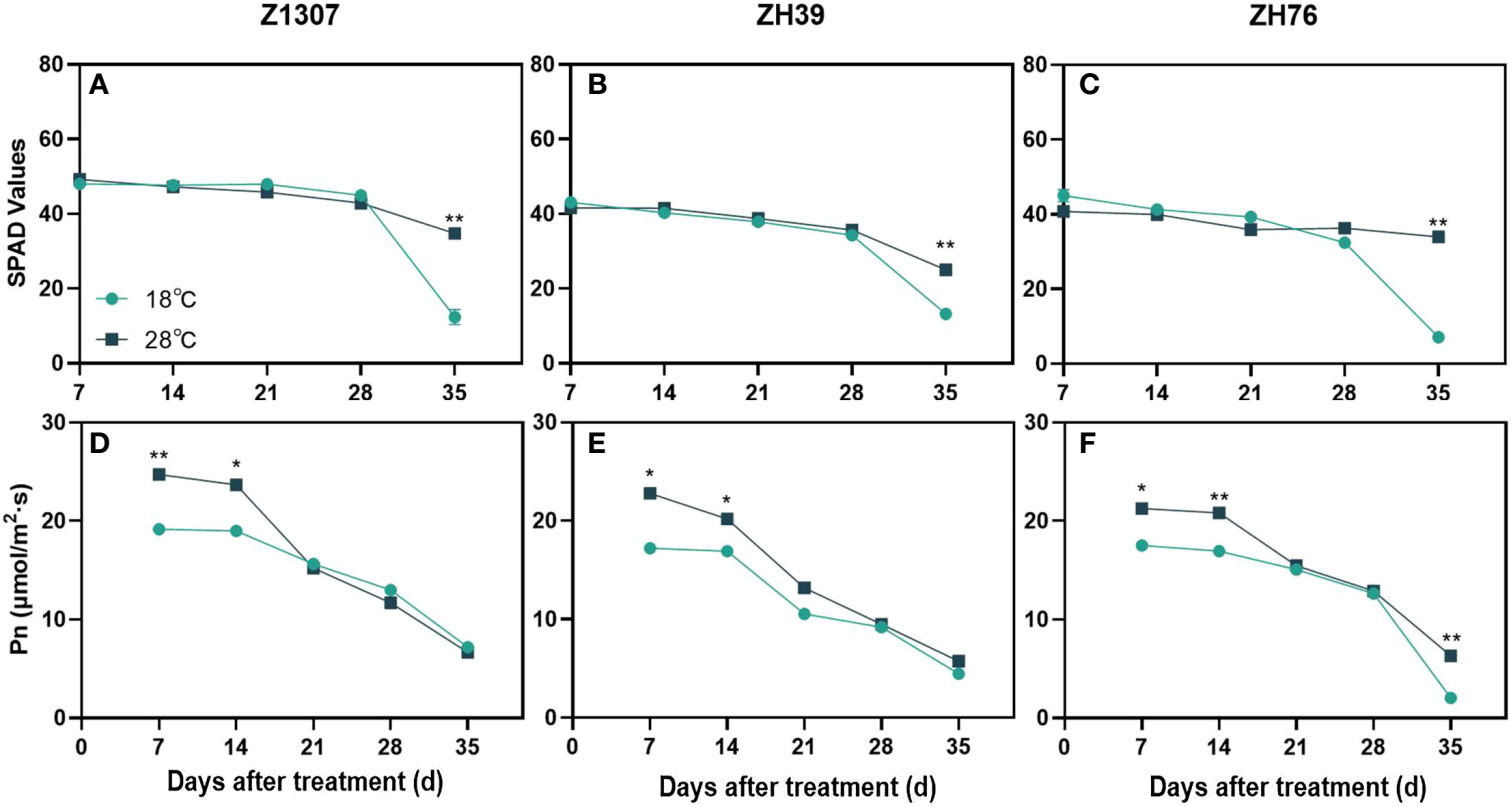
Effects of high night temperature on SPAD value and net photosynthetic rate of leaves under different night temperatures. The light blue/ black blue line indicates the 18 ℃/28 ℃ treatment. The broken lines respect the dynamic changes of the SPAD value **(A-C)** and photosynthetic rate **(D-F)** of soybean leaves with the days of different high night temperature treatments for the three varieties of Z1307, ZH39 and ZH76. Values shown are means ± SE from three biological replicates. (*, *P* < 0.05; **, *P* < 0.01).

There was no change of the SPAD value in both varieties until 28 DAT in the three varieties, while it was obviously raised under 28°C night temperature treatment at 35 DAT, indicating that high night temperature induced slight green-stay of the leaves (**Figures 4A-C**). The high night temperature significantly improved the net photosynthetic rate of leaves before 14 DAT (**Figures 4D-F**), while it was decreased gradually after 14 DAT in the three varieties. No significant difference in the net photosynthetic rate between the two treatments during 21 and 28 DAT in both varieties. In addition, no significant difference in net photosynthetic rate was found between the two treatments at 35 DAT in Z1307 and ZH39, while it was significantly increased in ZH76 at 35 DAT.

### 3.6 Sucrose and starch content

**Figure 5:**
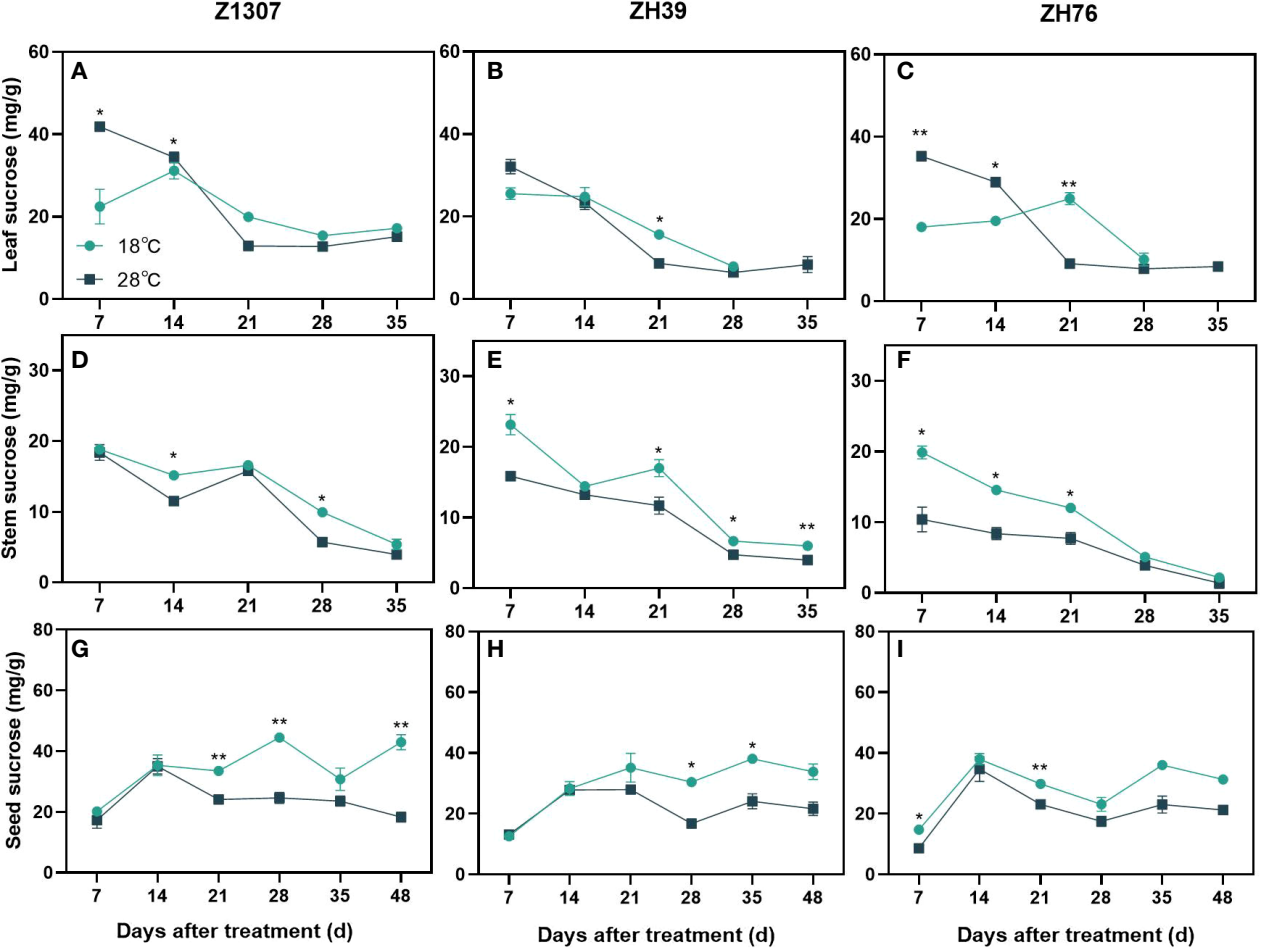
Effects of high night temperature on sucrose content in soybean leaf, stem and seed. The light blue/ black blue line indicates the 18 ℃/28 ℃ treatment. The broken lines respect the dynamic changes of the sucrose content of leaf **(A-C)**, stem **(D-F)** and seed **(G-I)** with the days of different high night temperature treatments for the three varieties of Z1307, ZH39 and ZH76. Values shown are means ± SE from three biological replicates. (*, *P* < 0.05; **, *P* < 0.01).

The dynamic change pattern of sucrose in stem and seed was similar under different night treatments for the three varieties, however, it was obviously different in leaf (**Figure 5**). The sucrose content in leaf was significantly higher before 14 DAT, while decreased rapidly during 14-21 DAT. The sucrose content in stem and seed of 28°C night temperature treatment was always lower than 18°C treatment. The three varieties showed the same trend, but the period of difference was not consistent.

**Figure 6:**
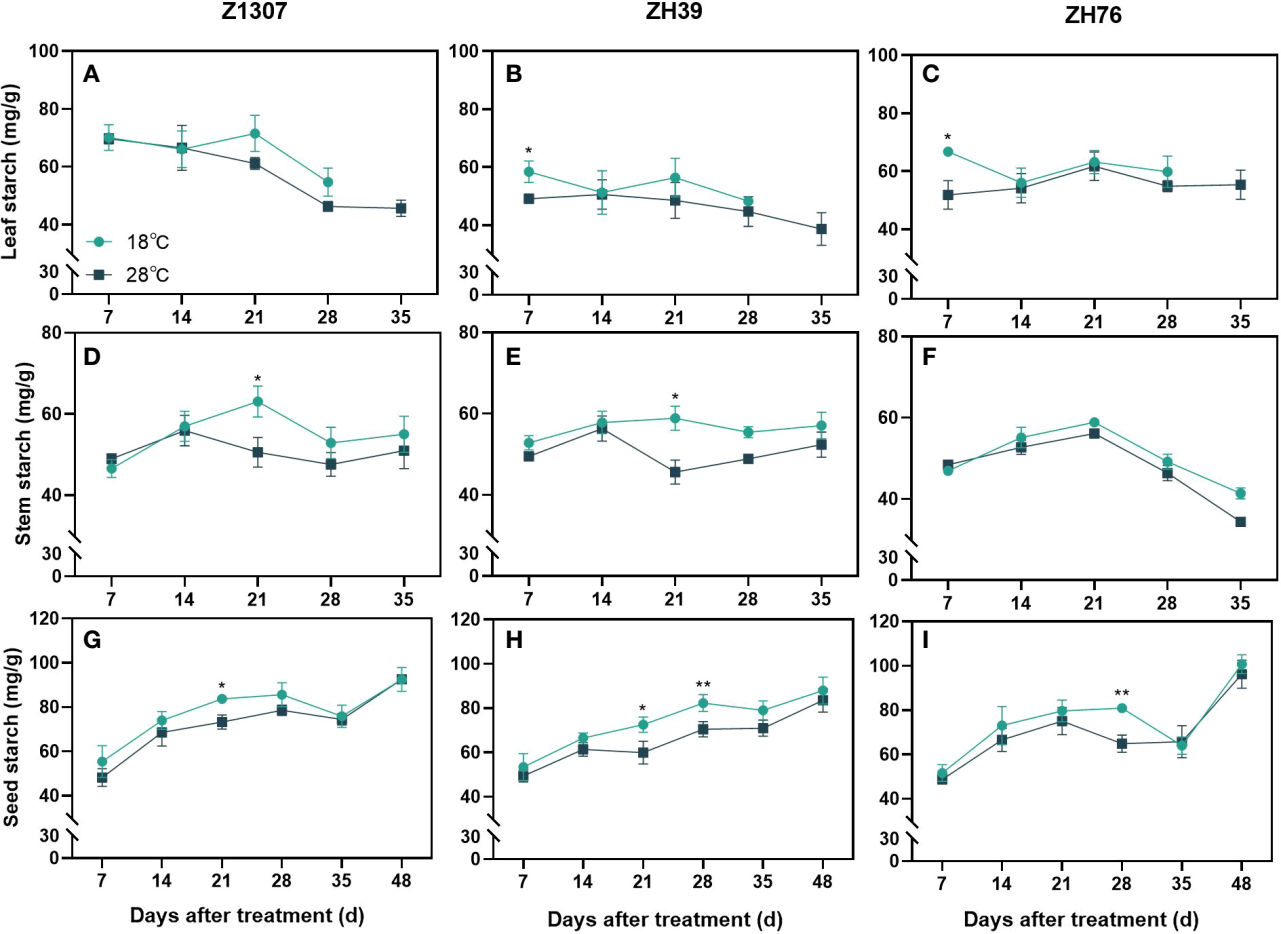
Effect of high night temperature on starch content in soybean leaf, stem and seed. The light blue/ black blue line indicates the 18 ℃/28 ℃ treatment. The broken lines respect the dynamic changes of the starch content of leaf **(A-C)**, stem **(D-F)** and seed **(G-I)** with the days of different high night temperature treatments for the three varieties of Z1307, ZH39 and ZH76. Values shown are means ± SE from three biological replicates. (*, *P* < 0.05; **, *P* < 0.01).

Leaf starch content in the elevated night temperature treatment was significantly higher in ZH39 and ZH76 at 7 DAT, and it showed no difference in Z1307 (**Figure 6**). For the three varieties, leaf starch content changed non-significantly after 14 DAT. Under the 28°C night temperature, stem starch content peaked at 21 DAT and then decreased, with significant differences in Z1307 and ZH39 varieties and no difference in ZH76. High night temperature improved the starch accumulation in the seed, with significant differences in Z1307 and ZH39 at 21 DAT, highly significant differences in ZH39 and ZH76 at 28 DAT, and non-significant differences at maturity between treatments.

### 3.7 Relationships between dry matter accumulation and yield per plant

**Figure 7:**
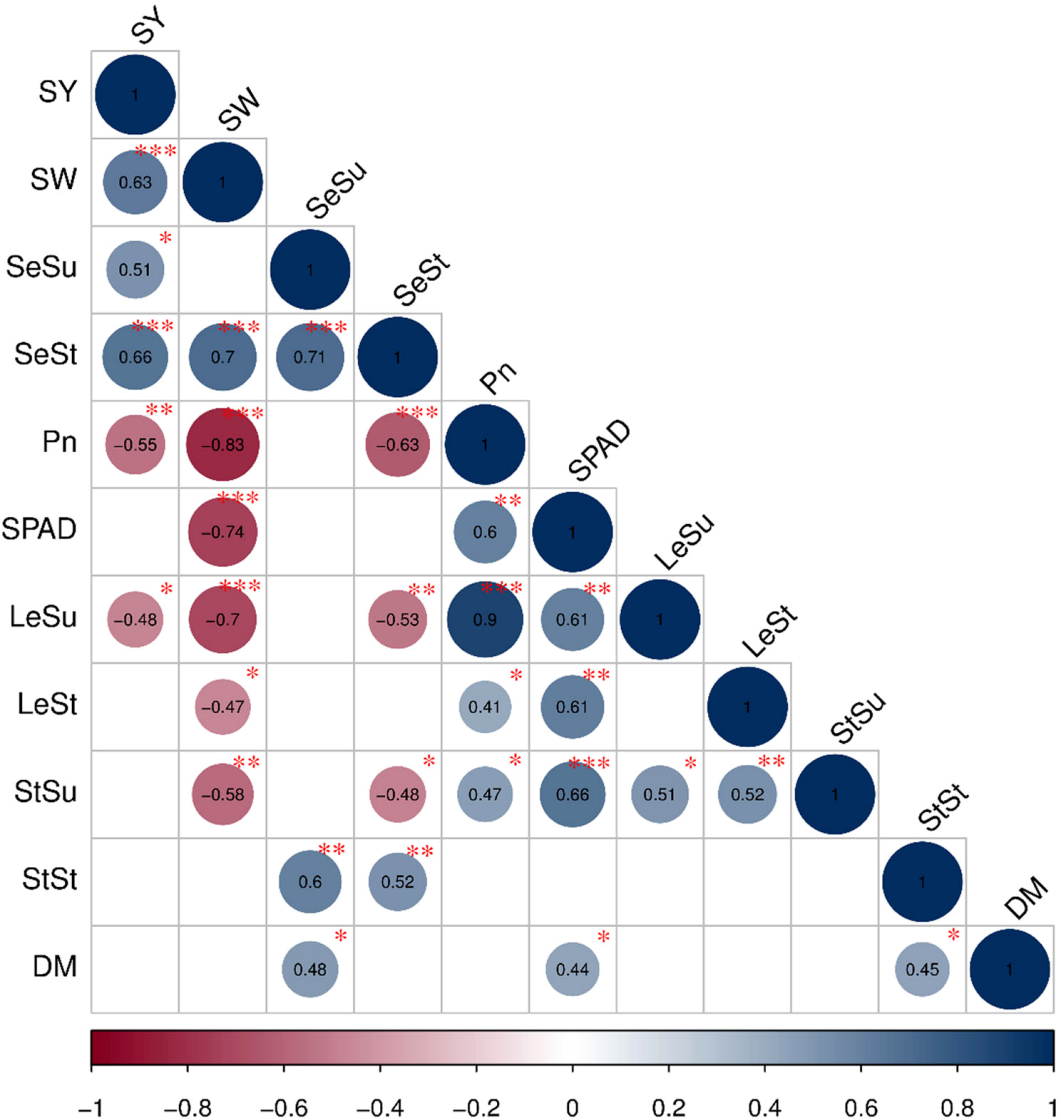
Pearson correlation analysis showing the relationships between growth factors and grain yield. The circle size shows the significant level and the circle color exhibited the positive or negative correlations. P-values less than 0.001, 0.01, 0.05 are indicated by the asterisks and dot symbols: ‘* ** ’, ‘* *’ and ‘* ’.The abbreviations used in the figure are followed as: seed yield per plant (SY), seed weight (SW), seed sucrose content (SeSu), seed starch content (SeSt), photosynthetic rate (Pn), leaf sucrose content (LeSu), leaf starch content (LeSt), stem sucrose content (StSu), stem starch content (StSt), dry matter per plant (DM).

As shown in **Figure 7**, single plant yield was positively correlated with single seed weight (*P<*0.001), starch (*P*<0.001) and sucrose content in seeds (*P*<0.05) and negatively correlated with both the net photosynthetic rate (*P*<0.01) and sucrose content in leaf(*P*<0.05). Single seed weight was positively correlated with starch content in seeds (*P*<0.001), and negatively correlated with the net photosynthetic rate in leaves (*P*< 0.001), the SPAD value (*P*<0.001), the sucrose content (*P*<0.001) and the starch content in leaves (*P*<0.05), and the sucrose content in stems (*P*<0.01).

### 3.8 Transcriptome analysis

A total of 1861 differentially expressed genes (DEGs) were identified between 18°C and 28°C treatment, 953 of which were down-regulated (**Figure S3** and **Table S1**). GO and KEGG analysis indicated that these DEGs perform a wide range function, but the most of them are involved in carbohydrate metabolism, transmembrane transport, and ion channel-related processes, photosynthesis, amino acid metabolism, biosynthesis of other secondary metabolites and biological clock (**Figure S4**). This analysis suggests that DEGs are associated with energy expenditure, ion uptake, transport, stress tolerance, oxidative stress and scavenging under high night temperature.

High night temperature regulated the major differential pathways of carbohydrate metabolism in soybean leaves, including 11 DEGs for photosynthetic carbon fixation (7 up-regulated and 4 down-regulated), 30 DEGs for carbon metabolism (14 up-regulated and 16 down-regulated), 19 DEGs for glycolysis/gluconeogenesis (7 up-regulated and 12 down-regulated), 19 DEGs for pyruvate metabolism (9 up-regulated and 10 down-regulated), 29 DEGs for starch and sucrose metabolism (8 up-regulated and 21 down-regulated), 16 DEGs for amino and nucleotide sugar metabolism (5 up-regulated and 11 down-regulated), 5 DEGs for pentose phosphate pathway (1 up-regulated and 4 down-regulated), and 6 DEGs for fructose and mannose metabolism (3 up-regulated and 3 down-regulated).

**Figure 8:**
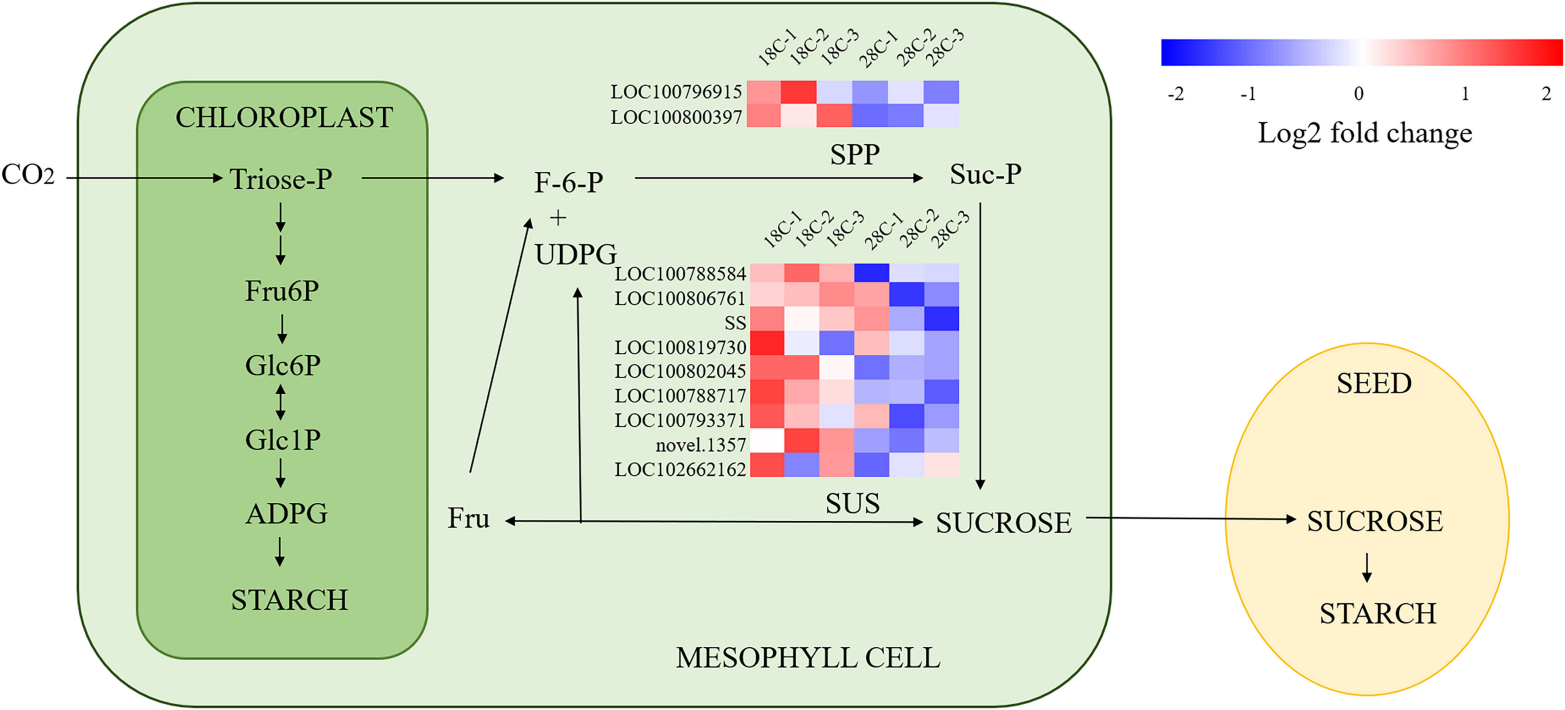
A simplified version of the starch and sucrose metabolic pathway. Heat map indicates the Differential Expression Genes (DEGs) associated to the starch and sucrose metabolic pathway. Red indicates up-regulated genes, and blue indicates down-regulated genes. The pathway is modified from a figure in ref (Fünfgeld et al., 2022).

It was found that among the DEGs for sucrose metabolism, 7 of 8 sucrose synthase genes and 2 Sucrose-phosphatase genes were down-regulated, including LOC100802045(Glyma.19G212800), LOC100788584 (Glyma.17G045800), LOC100788717 (Glyma.15G151000), LOC102662162 (Glyma.03G048351v4), LOC100806761 (Glyma.09G073600), LOC100793371 (Glyma.03G216300), SS, LOC100819730 (Glyma.15G182600), LOC100800397 (Glyma.10G086600), LOC100796915 (Glyma.20G070500), implying the sucrose synthesis of leaves was regulated at the transcriptional level by high night temperature at 7 DAT (**Figure 8**). Among the photosynthetic carbon fixation-related genes, *RBCS2*, which catalyzes the reaction of fixing CO_2_ in photosynthesis and is an important enzyme related to chloroplast function (Xing et al., 2022) is found up-regulated, which may be related to the rise of the photosynthesis at 7 DAT in this study.

## 4 Discussion

Soybean is sensitive to light and temperature. When subjected to photothermal change, it must constantly employ adaptive mechanisms to maintain homeostasis in photosynthesis, carbon fixation, and photosynthetic production (Pommerrenig et al., 2018). These adaptive mechanisms can both ensure the normal growth of the soybean but caused changes of biomass accumulation, individual development, material partitioning, and yield (Du et al., 2020). A systematic understanding of the relationship between elevated night temperatures and yield-related physiological indicators is beneficial for improving field cropping strategies and achieving improved soybean yield and quality.

The negative effect of high night temperature on soybean yield was reported in previous studies (Egli, 2010; Nakagawa et al., 2020), and it was consistent with the results of many other crops such as rice (Bahuguna et al., 2016, Welch et al., 2010), wheat (García et al., 2015; García et al., 2016), and cotton (Loka and Oosterhuis, 2010). It was found that high night temperature increased respiration-driven carbon losses and hindered the transport of photosynthetic assimilation products from source organs to reservoir, resulting in inadequate seed filling and ultimately a reduction in yield, which is consistent with the study of Tacarindua et al. (2013). The source-sink relationship during grain development was affected by temperature was also studied in many crops, such as rice (Arshad et al., 2017) and wheat (Hein et al., 2022). The negative correlation of soybean yield and quality under high night temperature reported in previous study (Choi et al., 2016) was confirmed in our study. It has also been reported that high temperature may affect seed weight by altering protein content (Nakagawa et al., 2020). A “concentration effect” resulted from a decline in the proportion of carbohydrate and seed size, caused the increase relative protein and oil content was also observed in our study. Furthermore, the protein and oil yield of individual soybean plants was found reduced under high night temperature, implying the high night temperature affects the accumulation of different components, but it had more effects on the carbohydrates than proteins and oils.

In this study, 28°C high night temperature treatment led to an increase in net photosynthetic rate in early 7 DAT functional leaves, contrary to the results of some studies of heat stress (Jumrani et al., 2016; Prasad et al., 2017; Ferguson et al., 2021), this may as a result of treatment carried out in the night and the 28°C night temperature was a mild heat stress for soybean (Hatfield et al., 2011). Some studies investigating the effects of night temperature concluded that there was no effect on net photosynthetic rate (Zhang et al., 2020), which was also different from our results. This may be due to the differences of treatment and crop species. Previous studies have reported that soybean leaves senesce later in controlled temperature treatments with 1-3°C higher environmental temperature (Tacarindua et al., 2013). This is consistent with the findings of our study and the stay-green of the leaf could be the reason for the decrease translocation of accumulated photosynthetic assimilation products in the leaves and the weakened efficiency of the photosynthetic product utilization.

Elevated levels of sucrose occurred early in this study, which may be related to the compensation for the carbon starvation caused by its accelerated degradation at night (Peraudeau et al., 2015; MacNeill et al., 2017). After a period of treatment, the sucrose content begins to remain relatively stable as it adapts to the change in temperature. The starch content in stems and leaves was found to be lower than the control under the high night temperature treatment after 21 DAT. The decrease in sucrose content in the seeds partly explains the decrease of the residue in seeds. Similarly, in rice, it was found that one of the causes for grain stunting under heat stress during reproductive growth was the restricted carbohydrate availability, and that heat stress reduced the movement of photosynthetic assimilation products into the seeds and inhibited the accumulation of starch in the seeds (Arshad et al., 2017).

The identified DEGs for photosynthetic and carbohydrate are associated with early increased sugar accumulation in the leaves treated under high night temperature treatment. Photosynthesis could be improved by the increased carbon fixation and sugar metabolism. Also, leaf senescence triggered by sugar accumulation leads to increase of nitrogen content (Wingler et al., 2006, Kaschuk et al., 2010). DEGs related to the transported nitrogenous compounds glutamine and glutamate were identified (Masclaux-Daubresse et al., 2010). Some amino acids or organic acids, such as proline and glutamate, are synthesized in large quantities when plants are subjected to heat stress (Vergara-Diaz et al., 2020; Wahid, 2006), and we also find the DEGs for these compounds. The transcriptome results reflect the response mechanism to high night temperature treatment conditions at the beginning of the seed filling stage. Some previous studies have suggested that changes in seed size and quality under heat treatment conditions could be associated with the expression of some heat-stimulated proteins (Kumar et al., 2015; Kumar et al., 2016; Kumar et al., 2020; Tiwari et al., 2021). Most HSP-related genes showed down-regulation in our study (**Table S1**), it could be explained by the mild stress caused by the moderate high night temperature we used.

This study expounds on the current understanding of the relationships between photosynthesis, plant development, carbon and nitrogen transport, and metabolism under high night temperature conditions. In addition to exploring response and tolerance mechanisms at the molecular level, we also need to optimize crop management strategies to improve soybean plants’ resistance to nighttime warming. In the context of global warming, breeding soybean varieties with low respiration and high energy efficiency is one of the most important ways to effectively address the world food crisis.

## 5 Conclusion

This study indicated that high night temperature during the soybean seed filling period (R5-R7) reduced seed yield and changed the proportion of the seed compositions. The decline of carbohydrates in seeds was the primary reason for the reduction in yield. The dynamic changes of dry matter accumulations, non-structural carbohydrates, net photosynthetic rate and SPAD value, as well as the transcriptome analysis explained the adaptive mechanisms for soybean to high night temperature stress at physiological and molecular level. The study is of significance for improving soybean breeding and cultivation practice to fight the global warming.

## Data availability statement

The original contributions presented in the study are publicly available. This data can be found here: NCBI, PRJNA892245 and SAMN31361599-31361604.

## Author contributions

LY and WS conceived the initial research plan, conducted the experiments, and analyzed the data. CW supervised the research and provided the laboratory infrastructure and funding. LY prepared the graphs and wrote the article. CW, DJ, WS, CX, and ES critically reviewed and edited the manuscript. All authors contributed to the article and approved the submitted version.
