# Supplementary figures and images for: Effects of high night temperature on soybean yield and compositions
Source: Front Plant Sci. 2023 Feb 17;14:1065604. doi: 10.3389/fpls.2023.1065604 (PMC9987466; doi:10.3389/fpls.2023.1065604)

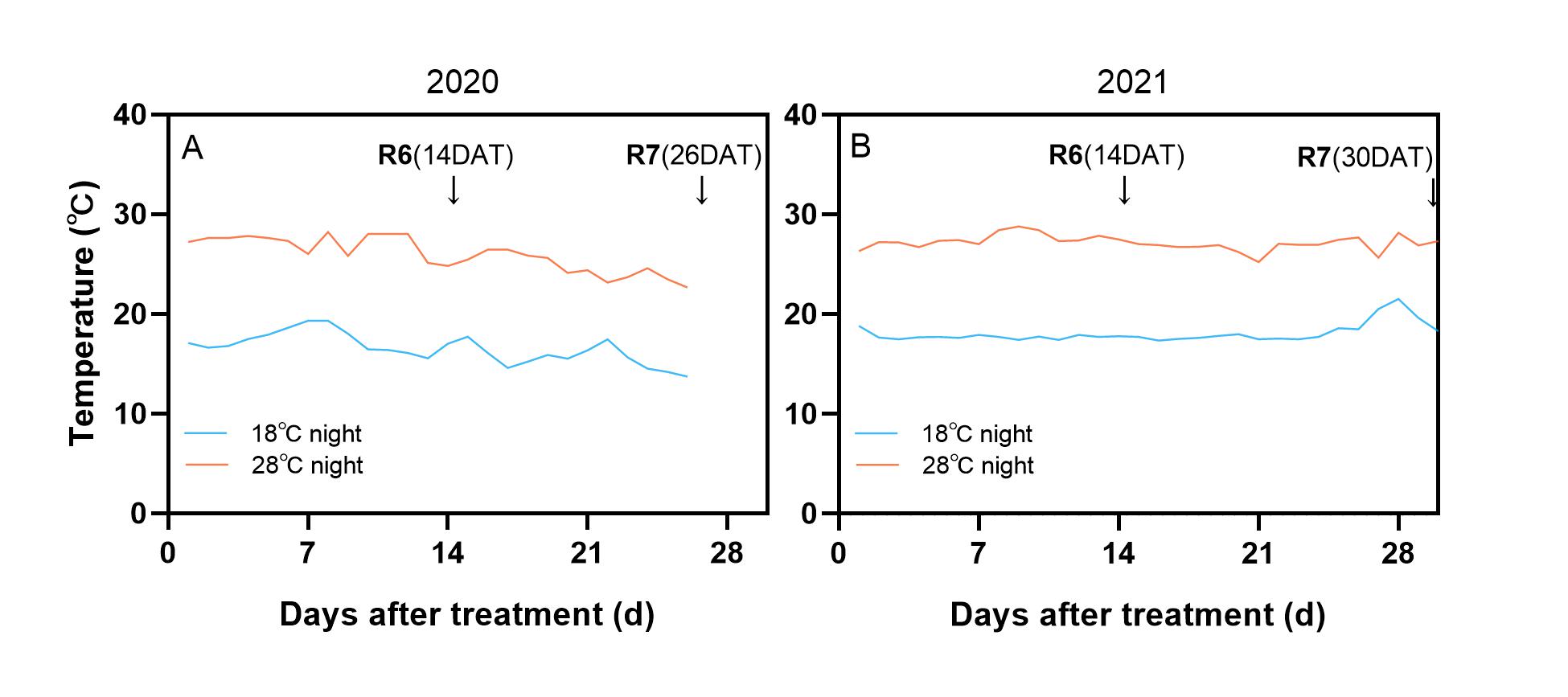

Supplement: Supplementary file 2 [file Image_1.jpeg]

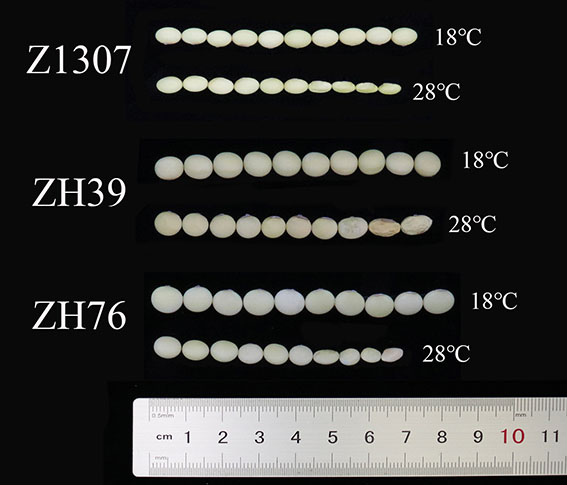

Supplement: Supplementary file 3 [file Image_2.jpeg]

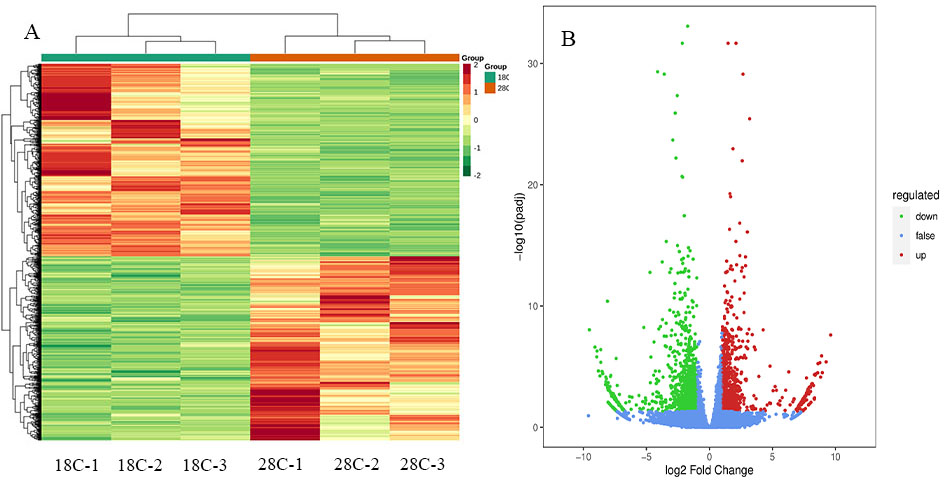

Supplement: Supplementary file 4 [file Image_3.jpeg]

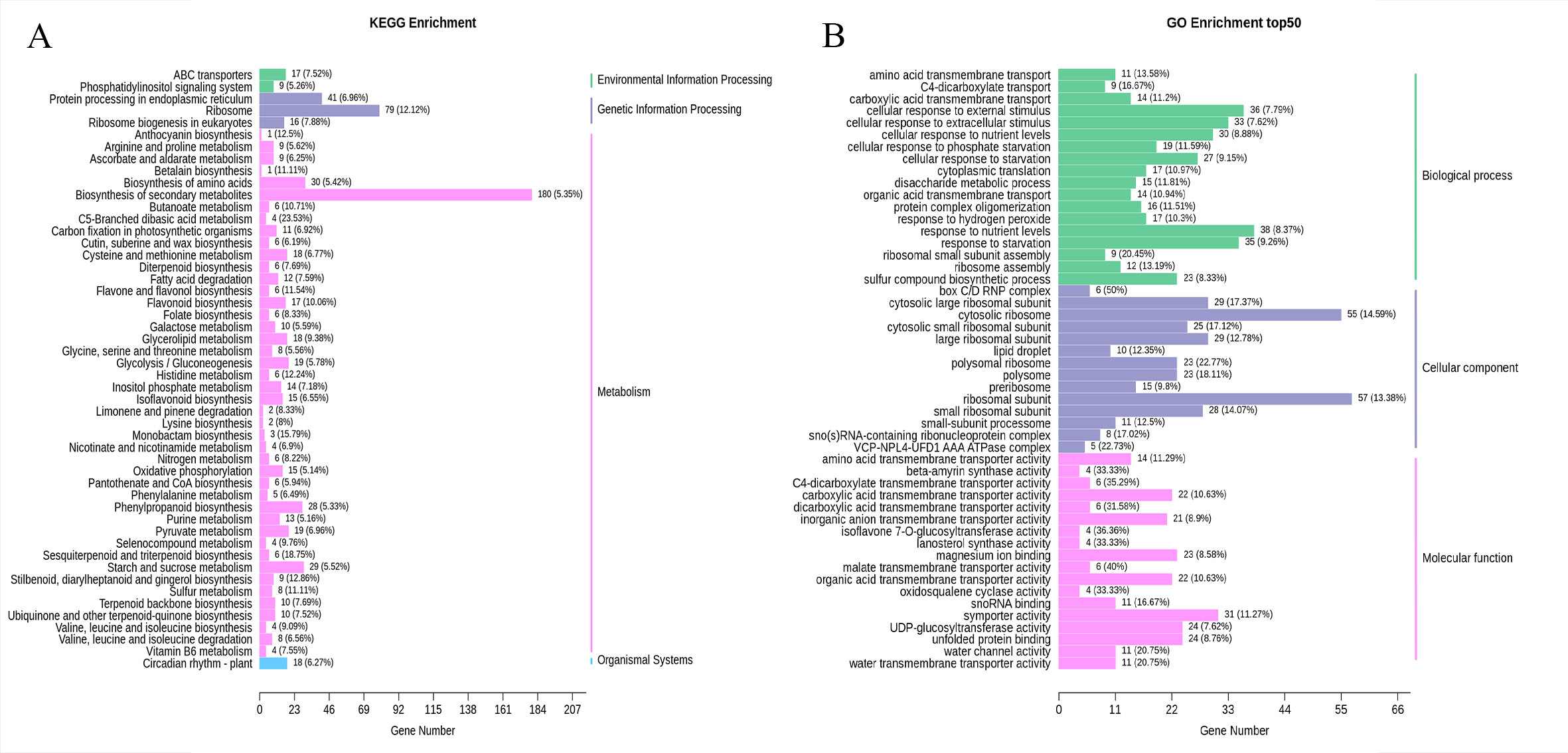

Supplement: Supplementary file 5 [file Image_4.jpeg]
